# Adaptive Relay Free Space Networking for Autonomous Underwater Drone Swarms

**DOI:** 10.3390/s25247412

**Published:** 2025-12-05

**Authors:** David Stack, Douglas Nuti, Mehdi Rahmati

**Affiliations:** Washkewicz College of Engineering, Cleveland State University, Cleveland, OH 44115, USA; d.m.stack12@vikes.csuohio.edu (D.S.); d.a.nuti42@vikes.csuohio.edu (D.N.)

**Keywords:** underwaterwireless optical communications, underwater autonomous drones, swarm robotics, cooperative communications, networking protocols

## Abstract

Underwater wireless networking is an emerging field for exploration and monitoring, enabling real-time data transmission and communication with both static sensors and submersibles. Current approaches mostly focus on utilizing acoustic waves. The use of optics for this purpose has been known to have several implementation challenges that have prevented it from being considered as a universal alternative. This study proposes that utilizing optics in an adaptive relay wireless network configuration can overcome its primary limitation of line-of-sight (LOS) propagation. In this paper, a network of strategically placed sensors is experimentally constructed with the ability to read and send modulated blue light, fit for extended submersion in water. This proposal represents a hypothetical aquatic drone swarm that is developed and programmed to follow adaptive relay logic. This network is able to demonstrate adaptation to obstructions in the LOS and maintain communication through configurations in which the sender and intended recipient would otherwise be unable to directly communicate. This finding allows the advantages of optical communications to be further explored for aquatic applications, primarily its higher potential data rate, which is inherently productive to a swarm.

## 1. Introduction

Underwater autonomous drones are an emerging field with many unanswered questions in motion planning, navigation, power management, and reliable communication and networking. Current well-developed radio-frequency-based wireless methods used in surface or unmanned aerial drones, such as cellular, satellite, Wi-Fi, long-range radio (LoRa), are practically unusable underwater [1,2,3,4]. There are two primary ways that the limitations on current wireless communication methods imposed by an underwater environment are overcome: acoustic communications [1,2,3,4,5,6] and optical communications [1,2,3,4,5,6,7]. Acoustic methods, including acoustic modems, Sonar, ultrasound communications, remain the primary means of underwater long-range communications due to the properties of acoustic waves [2,4,5,6]; however, they have a lower bandwidth, data rate, and longer propagation delay that may prove problematic when trying to coordinate movements in a swarm [6]. While a hybrid acoustic-optical approach is often recommended [2,4,5], optics have distinct advantages that make it desirable for swarm coordination [1,2,4,5,7].

Optical methods, including visible light, laser, ultraviolet (UV) also face challenges underwater, including color-dependent attenuation depending on water conditions, line-of-sight dependencies, and a comparably limited range [1,2,3,4,5,7]. While most of the color problems are solved by using light with a wavelength in the blue or green range [1,2,4,5], it is the other limitations that are addressed by using a swarm. Current approaches using optical communication either use optics only to attempt to mimic natural swarm formations [1,2,7] or rely on acoustic/hybrid methods for communication over pure optics [3,5,8]. Organizing an optics-based intelligent swarm (as opposed to a mimic-nature swarm) can be used to extend the limited range and compensate for the line-of-sight limitations, enhancing the effectiveness of wireless optical systems.

Current limitations of free-space optical communication systems can be addressed by integrating acoustics into a hybrid opto-acoustic communication system [2,4,5]. However, this approach presents challenges related to cost and hardware implementation due to the complexity of designing a dual-waveform system, which may not be feasible in all scenarios or robotic designs. To make a swarm intelligent, forming a network is necessary. Networking in a swarm is its own challenge, both in and out of water. Current swarm networks either only attempt to mimic natural swarms [1,3,7,9,10] or rely on communication methods that are not usable underwater [10,11]. Advancements in algorithms that focus on independently and intelligently completing complex tasks are being made for above-water swarms [11], but an optics-based wireless underwater system that implements similar algorithms is not currently readily available.

**Figure 1:**
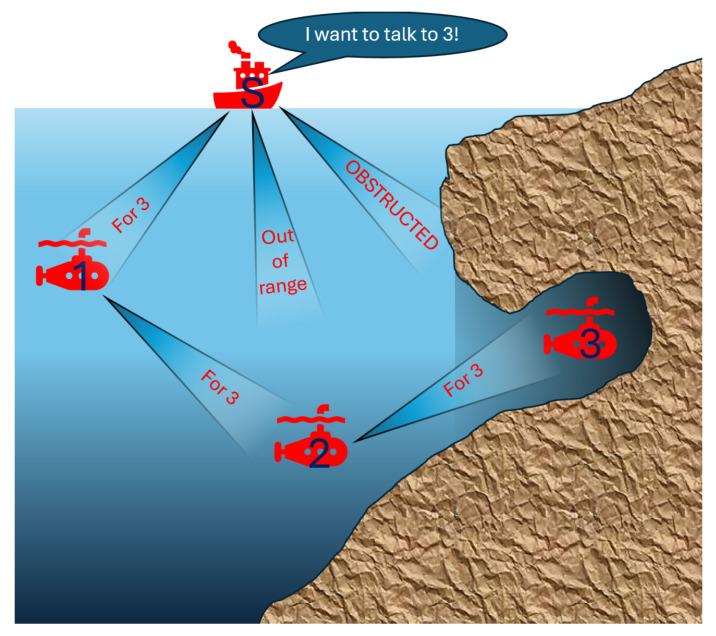
The proposed system model including multiple underwater drones and a surface drone communicating using optical waves.

This paper proposes an underwater environment-specific wireless cooperative optical network. It examines the prototyping and practical steps involved in experimentally testing a network of underwater drones that utilize wireless optical communications for networking and swarm operations. The line-of-sight nature of such a system necessitates that the network formed be able to determine possible configurations independently. Additionally, each part of the swarm must be able to receive instructions regardless of whether that member has a direct LOS to the member giving the instructions. The creation of such a system would facilitate underwater exploration for maintenance, environmental monitoring, search and rescue operations, and mapping, making it easier to access and create a safer, cleaner underwater environment. Additionally, the lack of a tether would enable these benefits to be usable in tight spaces and near propellers. Although each individual drone has a shorter communication range than currently available wired drones, a wireless drone system is theoretically infinitely extendable through relays. This is a potential door-opening step for practical underwater monitoring systems. Both aquatic optical wireless communication and adaptive relay communication have been developed previously; a network designed to accommodate both is unexplored. The aim is to develop a submersible adaptive relay optical wireless network. Figure 1 shows a schematic of the proposed solution, showcasing a cooperative communication where nearby nodes act as on-demand relays to route around LOS blockages, which results in near-continuous connectivity and higher overall throughput under dynamic occlusions. This will be discussed in more detail in the following sections.

The rest of the paper is organized as follows. Section 2 describes the benefits and limitations of the free-space underwater optical communications. Section 3 covers the electrical, physical, and network construction and considerations made for this system, as well as the experimental methods used to prove its capability. Section 4 displays each part of the experiment as well as the data obtained from the experiment. Also found here is the analysis of the results and the conclusions that can be drawn from them. Section 5 concludes the paper, discusses the interpretation of the results, and provides suggestions for future planned implementations of this system.

## 2. Theory and System Model

Free-space optical communications systems are generally seen as vulnerable to scattering and absorption [2,3,4,5,6,12,13,14,15,16]. Both are forms of signal attenuation caused by several conditions. Scattering is a limitation of wave-based communication systems caused by the original wave reflecting and refracting through any number of optical obstacles or differences in the medium of its channel [14]. This effectively causes the signal to become more sporadic the further away from the source it is received, reducing the distance at which a signal can be reliably detected. Absorption is a phenomenon related to the turbidity of a medium [16]. The turbidity, i.e., lack of clarity of the water, results in a decline in the amount of light that can pass through. These forms of attenuation are unavoidable in water and can only be compensated for. The underwater optical channel can be modeled using the Beer-Lambert law [17], in which the received optical power decays exponentially with distance, such that $P_{r}\left(d\right)=P_{t}e^{-c(\lambda )d}$. Here, c(λ)=a(λ)+b(λ) represents the wavelength-dependent extinction coefficient a(λ) and and the scattering coefficient b(λ). Scattering in the visible range is explained by Mie theory due to the particle-size distribution in natural waters, and typically dominates in coastal or turbid environments. This exponential attenuation model is widely used in underwater optical wireless communication.

This study aims to address the challenges of the other known limitations and compensate for the effects of scattering and absorption by integrating an adaptive relay swarm network into a free-space optical communication system. This type of cooperative communications system is known for its range, reliability, and ability to overcome blockages [6,18,19,20]. These advantages will allow for the other known limitations facing optical systems in an aquatic environment to be addressed, including:

- Line-of-sight dependent [2,4,9,12,13,18,19]
- Limited communication range [2,4,5,12,13,14,16]
- Vulnerable to channel obstructions [4,12,18]
- Vulnerable to light interference [4,12,15,19]

These issues prevent the distinguishing benefit presented by such optical systems, increased data rate, from prevailing. Implementing such a system as a swarm using an adaptive relay network can overcome these issues in a way that does not dramatically sacrifice the higher data rate, as is the case with current hybrid acoustic solutions.

Hybrid acoustics solutions seek to address the known limitations by using acoustic communications either in tandem with optics or whenever optics fail. If used in tandem, the majority of the system’s maximum data transfer rate will depend on the optical part of the data. If used where optics fail, the systems will be entirely limited by the data rate of the acoustic communications until optics can be restored. An adaptive relay swarm can address the limitations without sacrificing the data rate to the extent that acoustic solutions do.

In a swarm communicating as an adaptive relay network, the line-of-sight dependency can be greatly mitigated by relaying the data through other members. As long as a valid path is discoverable, the source does not need to have a direct line of sight to its destination. The greater the number of members in the swarm, the more possible ways to maintain segmented line-of-sight chains. The limited communication range can be overcome by using other members as a traditional relay. Even if the source is out of range with its destination, as long as there is an in-range unit that can act as a step gap to either yet another unit or the destination, communication can be maintained. Similar to the previous issues, obstacles can be overcome as long as a path can be made. Even facing a sudden visual obstruction, communication should be interrupted as long as it takes to switch to or discover a new valid path. Its vulnerability to light interference is an issue that can not be directly solved by an adaptive relay swarm, but in a case where not every unit is facing interference, communication can be maintained. In the case of aquatic communications, deep water has no significant natural light interference. Near-surface cases risk facing the sun as an unavoidable source of interference. Commonly available blue-enhanced photo diodes, when paired with narrow-band optical filters, can mitigate most of the unwanted wavelengths, which fall out of the desired spectrum. As for the DC interference from the remaining wavelength within the filtered range, thresholding techniques or AC/DC separation techniques at the photodiode front-end will mitigate the remaining interference.

The longer the chain that must be formed to maintain communication, the slower the message will be. Acoustic systems more easily overcome the limitations, but keeping the communications within optics can allow for a significantly higher data rate to be maintained. Such a system can improve the current system by allowing for independent, high-data-rate underwater communications in areas currently accessible by wired drones.

Table 1 presents a qualitative comparison of some of the prior work in cooperative communications in the underwater optical channel. These papers can be categorized under amplify-and-forward (AF), decode-and-forward (DF), best-relay selection, hybrid Acoustic/FSO (HAFSO) relaying, multi-hop, and AI-assisted approaches. While the majority of these papers focus on either theoretical analysis or physical-layer relaying strategies, our paper presents a different approach via its system-level empirical cooperative networking protocol implemented in underwater optical settings.

## 3. Materials and Methods

### 3.1. Electronics

This section describes the electronics that were developed or chosen throughout this study, as described in the following subsections.

#### 3.1.1. Light Emitting Diode

This research began by determining the known practical limits of other free-space optical networks [1,2,4,5,6,7,12,13,14,19]. Following this review, it was determined that a fast-switching blue power light-emitting diode was the best candidate to act as a transmitter. LEDs similar to the models used for this experiment have shown that their data rate limit was around 25 Mbps [4]. This represents the effective maximum possible communication speed using current LEDs of this type per LED.

The choice of blue LED-generated light (450 nm wavelength) was made for both practical and ethical reasons. This narrow band generation helps to make this light more distinct from ambient light. Bright blue light is generally considered not harmful to fish [28]. An LED’s inherently wide-angle emission was deemed favorable for the purpose of this system. Experiments show that green and blue lights generally have the least attenuation in water. This is due to the fact that other colors are more affected by molecular absorption in water, which causes a reduction in range [4].

A more significant number of choices had to be made regarding the driver for this LED. A driver that supports a switching speed capable of producing a 25 MHz signal at the power requirements of a power LED was developed. This design was a slight modification to the common constant current driver circuit [29].

#### 3.1.2. Photo Diode

A photo diode was chosen to be the main detector for light. This circuit would serve as the primary receiver. Its small size and availability make it great for this setup, as multiple sensors can be placed around a unit. The driver would be based on a conventional yet reliable transistor logic circuit [30].

The photo diodes chosen are blue-enhanced, meaning that despite the photo diode naturally responding to the full visible spectrum, when paired with a narrowband blue optical filter, all that remains is a narrow blue band which can be detected. In real environments, external sources of light are likely to interfere with optical communications. This filtering helps reduce any ambient light noise from interfering with this communication. The blue light generated by the accompanying LEDs will be detected where other light sources will not, and the narrow bands of both the LED and photo diode overlap.

#### 3.1.3. Microcomputer

A microcomputer was chosen over a microcontroller for this design. A Raspberry Pi Zero 2 W [31] was preferred for its small size, dynamic nature, cost-efficiency, and its support of operating systems. A microcontroller would have provided a more consistent platform for such a system, but ultimately, the available coding libraries made the microcomputer a more attractive option for ease of development, debugging, and built in peripheral compatibility for rapid prototyping.

Paired with the WiringPi [32] library, the maximum execution speed for a reading on a single pin was expected to be approximately 6 MHz. While this is the maximum possible communication speed for this system, a variety of factors lead to this speed being reduced. The waveform produced by this system was unusable for on-off keyed communication.

This, paired with a planned 5 sensors per underwater drone, would have to be reduced considerably to make room for 5 bits checked per 1 bit written. Further issues with the programming of the detectors would lead to an arbitrarily low data rate being chosen for this system. A final data rate of 10 kbps with an identical final frequency of 10 kHz was chosen for the ease of testing. This system is fundamentally capable of much greater speeds, but a clear and controllable signal is necessary to prove its function as an adaptive relay.

While a 10 kHz signal was used, the effective data rate is significantly lower than 10 kbps due to the fact that not every bit transmitted can contain useful data. This loss will be lessened in severity by future faster systems. The lower speed would provide the processors ample time to read and process data.

**Figure 2:**
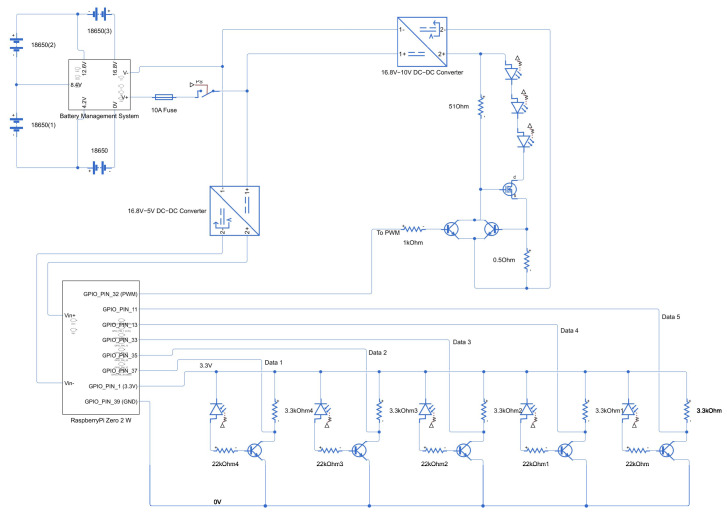
Full circuit. Note that the specific GPIO pins used for this design are largely but not entirely dependent on programming preference.

**Figure 3:**
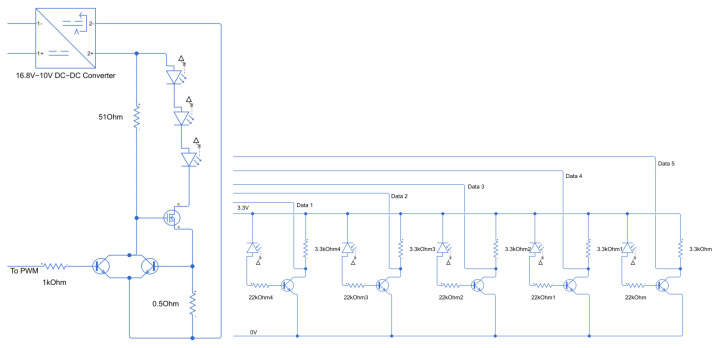
On the left is the LED driver. On the right are 5 repeated Photo Diode drivers.

The final circuit designed around the Raspberry Pi is seen in Figure 2. A more detailed close-up of the LED driver and the photodiode driver are seen in Figure 3.

### 3.2. Physical Construction

#### 3.2.1. Underwater Drone Body Design

Current commercially available systems, such as those made by BlueRobotics [33], exist for making custom aquatic drones. These systems present a high upfront cost in addition to not being explicitly intended for use in optical communication systems. In a swarm setting, the need for a scalable, low-cost solution is evident. We produced our own drone body in order to tailor it for the needs of this experiment, such as the clear body panel to allow proper light transmission while being unobstructed in most directions, as well as designs to allow the electronics to be housed without blocking light transmission.

**Figure 4:**
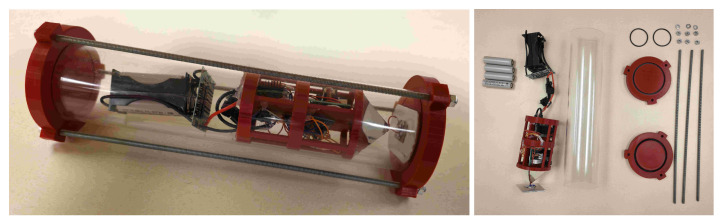
Designed prototype.

The body needed to be able to house all electronics with associated heat sinks, allow adequate light to pass through, and be able to support moderate pressurization. As such, acrylic was selected for the housing as it provided moderate strength under pressure while still allowing light transmission. The dimensions used are a 3-inch diameter and 14 inches in length, which provides ample room for future improvements. To seal the housings for the stand in drones as shown in Figure 4, a design consisting of two end caps with three thread-all bars was used, as the end caps contained rubberized gasket paper and an O-ring. To fully seal the housing, the thread-all bars are tightened down to create a full seal. For every researcher developed part 3D printing was used with a PETG+ filament choice. PETG+ was selected as it provided ample strength while having some elasticity to avoid any shattering or cracking that could be caused by compression, tension, or impact. With most 3D printing for purposes such as underwater drones, it is required to seal the print walls, as when 3D prints are introduced into pressurized underwater environments, water starts to leak through pores that form during the printing process, leading to leakage. To fix this issue, two methods were used: (i) liquid rubber spray and (ii) annealing with a heat gun. Both methods proved to be exceptional for sealing pores in 3D prints as the heat gun melted the walls, creating a smoother surface with fewer pores, while the rubber spray covered any remaining holes. For other iterations, an off-the-shelf plastic cement was used to further seal any problem areas, as the acetone contained in the product proved to semi-melt the surface layer of the applied location, creating a better filament seal while also hardening to provide further added protection.

**Figure 5:**
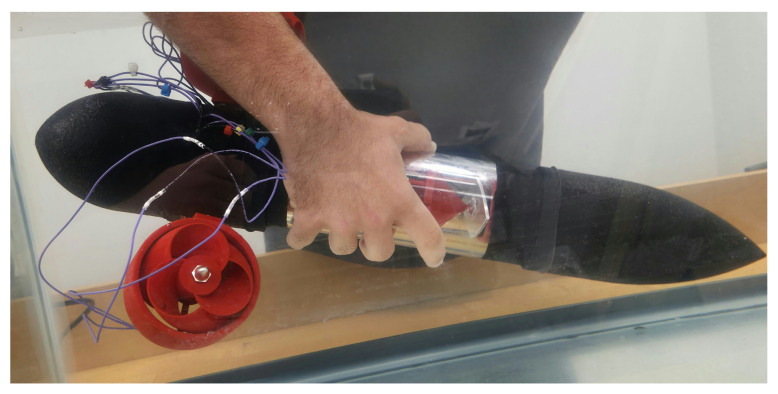
Movement capable unit in the water tank (Researcher partially blurred).

During the development of the stand-in drones, a movement-capable design was created to prove the capabilities of this technology in real applications. This was completed using O-ring pressure fittings for the front cone and outside pressure fittings for the back tail end. To help account for the lessened internal support in the tail end, the circuit mount had bulkheads, with the largest one being in the tail end. It is sealed with a combination of O-rings and latex. The tail end needed to be larger as it houses the servo motors and the motor control boards, meaning a design in which the tail end fits over the tube was utilized. The cone was designed to house the batteries and leave room for a front-mounted LED heat sink comprised of a cut 2-inch piece of 1/8 inch thick aluminum bar. The tail end was designed to house two servo motors used to turn the side body thrusters. The servos were made waterproof by removing the main covers and applying liberal coats of silicon-based waterproof glue, along with a coating of liquid electrical tape over any exposed electrical connections. Note, it is imperative to avoid any moving component with said sealants, such as the gearbox and the actual motor itself. Once the parts were printed, layers of a rubberized spray were added to further waterproof the main body, helping to reduce any leakage through the pores mentioned in previous sections. For the movement component, a combination of side-mounted servo motors with attached 920 KV brushless motors is used for propulsion with toroidal propellers. The added thruster housings were implemented to reduce any potential damage and increase output velocity by employing Bernoulli’s principle and decreasing the output radius to double the output velocity. This movable version is seen in Figure 5.

#### 3.2.2. Circuit Mounting Board

**Figure 6:**
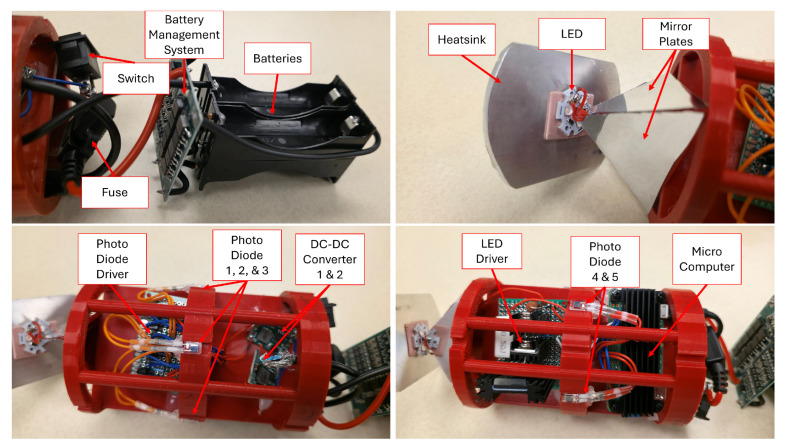
Components mounted within a mounting board.

**Figure 7:**
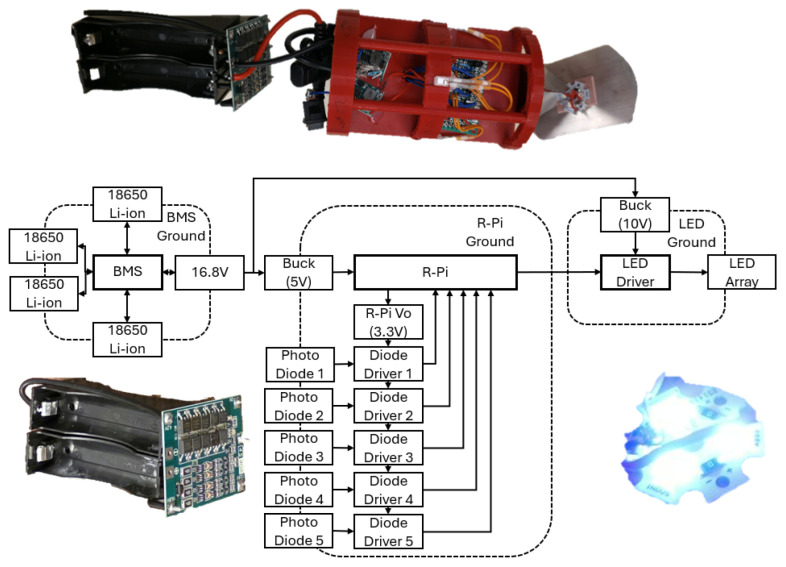
System block diagram of electrical systems.

For the submerged nodes, a circuit mounting board was designed. As the nodes needed to be able to secure any electrical component, provide ample field of view for both the photo detectors and the LED, be structurally sound, and allow room for wire routing. For mounting the electrical components, an initial board size of $2.74^{\prime \prime }$ by $4.51^{\prime \prime }$ with a thickness of $0.15^{\prime \prime }$ to allow the components to be attached on either side of the board while still providing space to route wires between the side of the plate and the acrylic tube. As this study relies on the ability of a node to send and receive signals, a pyramid of mirrors was integrated into the design of the circuit mounting board to allow the LED to display past the 180-degree limit. The mirrors protrude from the end of the circuit plate $1.49^{\prime \prime }$ at an interior angle of 45 Degrees. For receiving, 5 photo diodes are placed into a central ring integrated into the circuit mount, this ring is recessed to a diameter of $2.90^{\prime \prime }$ with cut outs every 72 degrees with dimensions of $0.21^{\prime \prime }$ width and $0.08^{\prime \prime }$ depth to account for the thickness of the photo diodes and allow them to be as close to contact with the acrylic tube for optimal viewing angle of each sensor while not restricting the side view, excluding directly in front or behind the node as this design excludes any photo detectors on the direct front or back. The number of photo diodes was chosen to increase the viewing angle of the drone to comfortably detect light within a 360-degree radius around it, and to give the system jam and cross-talk resistance. As this system relies on the careful use of a single physical channel, adding resistance to the system for such cases in which this single channel is not properly used is a priority. Having multiple channels allows for cases in which one or more photo diodes are effectively jammed by a noisy environment or cross-talk, and yet the unit’s ability to communicate at other angles persists. Structural stability is addressed by including two rings with diameters of $2.99^{\prime \prime }$ to ideally be in contact with the inner diameter of the acrylic tube to act as bulkheads, with the ring located in the back being larger in length to provide increased support. To allow wires to route along the outside of the rings, three cutouts with a radius of $0.10^{\prime \prime }$ were made along the rings to create cleaner wire management. This design does have minor issues, such as not accounting for the forward or backward detection, as well as not providing ideal display angles for the LED. Figure 6 shows all the components mounted to the mounting board. Figure 7 shows a system block diagram depicting each electrical system within a drone.

#### 3.2.3. Buoy Design

A cross-media method of communicating is needed to take drone-based communications and allow them to be received in the air. Light can inherently cross the water-air boundary; however, a means to translate the optical signal to a more accessible form (such as radio) will simplify above-water control of the system. Thinking of the means by which the light pierces the water as a buoy is key, as the light is best if it does not have to shine from above the water and has to penetrate its surface. The use of this means is not strictly necessary, but requiring the light signal to be properly interpreted after having crossed the boundary is an avoidable potential source of error, although possible. Sunflower, the first-of-its-kind system, demonstrated the feasibility of direct air-water localization using laser light between an aerial drone and an underwater robot [34].

Any above-water vessel or platform that can communicate with the user and allows an emitter to dip into the water to make clear its commands is a fitting stand-in for the buoy. As long as this stand-in can also read the signal being sent back to it, it is sufficient. A rope with an LED at the end of it dangling off a boat is another way it can be thought of. The buoy only needs to be able to send and receive signals, meaning no specific design is required, only a design that can remain within the stable view of the underwater drones.

### 3.3. Networking

#### 3.3.1. Protocol

Once the system was assembled with its established 10 kHz speed, a non-return-to-zero on-off keyed modulated communications (NRZ-OOK) packet was developed, consisting of 23 bits per packet. It starts with a 10-bit signature to denote the start of a message, then a 2-bit identification, followed by a 2-bit destination. The signature was chosen to be a unique pattern, making it so that no later part of the message could be confused as a second message. If the destination matches the receiving unit’s unique ID, it will continue to decrypt the message. From there, a 4-bit section denotes a standard set of communications for varying purposes. These can be expressed or thought of as the following:

- PING
- Look for ID 0 (B)
- Look for ID 1
- Look for ID 2
- Look for ID 3
- Enter relay mode
- Exit relay mode
- Affirmative
- Denied
- Who can be seen?

**Figure 8:**
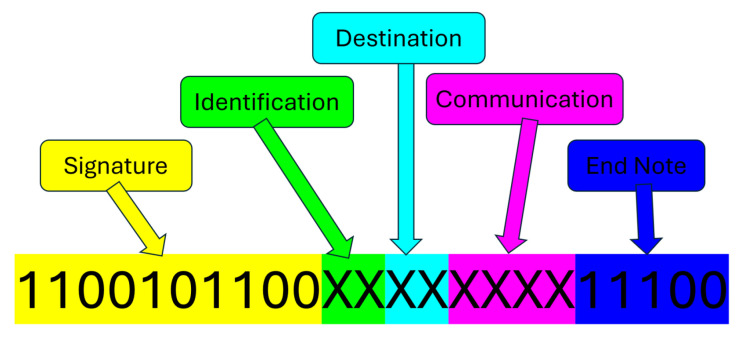
Breakdown of each section of the data packet.

After the message, a 5-bit end note was added to mark the end of the message. If any part of either the signature or the end note was missing, the communication attempt was considered invalid. No error correction was implemented for any data bit corruption for this system. Figure 8 shows a breakdown of the message packet.

While this set includes communication tailored for searching specific units, a scalable command for this purpose would be preferred but was not used in this study. This logic is dependent on a unique command for searching for each unit; however, systems that include greater than 4 bits for commands will see no issue in making room for such an expanded number of commands or a scalable alternative command. In any system of this nature, each unit must contain a unique ID in order to be addressed separately.

Most of the messages have a specific response tied to them. **PING** is the basic communication attempt. If any unit sees it, it will respond to it with **Affirmative**. For any of the look commands, a unit that receives it will verify whether or not it has a connection to the particular unit. The unit with ID 0 was reserved to act as the buoy and is otherwise referred to as unit **B**. It will transmit a **PING** message destined for the associated unit, and if it receives an **Affirmative** from that unit, it will itself return an **Affirmative**. If the attempt times out, it will return a **Denied**.

**Who can be seen?** is special. Whichever unit sends that message is coded to interpret the next received communication differently. It will instead use the 4 message bits as indicators of presents. The unit will fill in those bits denoting any unit it believes it can communicate with.

As should be expected, in an open-ended swarm, there is no guarantee that a message has a valid destination or is fully received. If nothing happens for too long, the unit will PING all IDs hoping for a response. No error correction was implemented in this setup.

#### 3.3.2. Adaptive Relay Logic

The proposed algorithm for node discovery and relay control is shown in Algorithm 1.
**Algorithm 1** Node Discovery and Relay Control Procedure   1:**PING** the surrounding network nodes.   2:**for** each node ID i∈{0,1,2,3} **do**   3:    **Check** if node with ID *i* is present.   4:**end for**   5:**if** relay mode is required **then**   6:    **Enter** relay mode.   7:**else**   8:    **Exit** relay mode.   9:**end if** 10:**Send** response: Affirmative or Denied. 11:**Query:** “Who can be seen?” to update visibility information.

**Enter relay mode** and **Exit relay mode** update an internal flag within a unit. When updated to true, a unit will note who has asked the unit to enter relay mode. The behavior of how the unit will treat incoming messages changes when in relay mode. A unit will still respond to messages intended for itself normally, but any message that is sent by the unit that originally sent the enter relay mode message (other than messages from that unit intended for the relaying unit) or any received message intended for the unit that triggered relay mode will be repeated with an edited ID.

**Figure 9:**
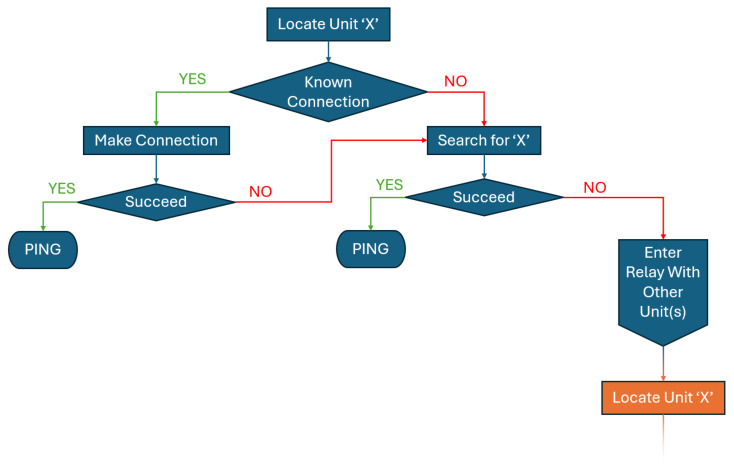
The proposed flowchart for a units search. The green arrows represent a move towards a successful communication. The red arrows represent a need to continue searching in order to communicate with the desired unit. The blue boxes represent all that can be done within one units attempt to communicate. The orange box shows the recursion point at which the search starts over but through a relay connection with another unit.

This changed behavior will continue until the **Exit relay mode** message is received from the triggering unit and intended for the relaying unit. The buoy is the primary unit in control of the systems and will make most of these requests, but every unit is capable of asking another to enter relay mode in order to determine possible connections. Should one branch of the search end without finding the destination unit, if there are any units visible to the searching unit but unchecked within that branch, the unit will proceed to ask those units until all possible communications have been exhausted. The proposed flowgraph is shown in Figure 9.

**Figure 10:**
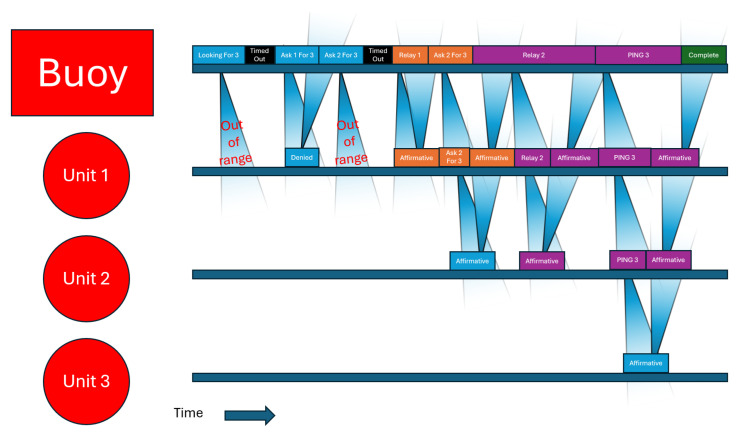
Timeline per unit of a long search. In this scenario, the only available communication paths are sequential node B can reach node 1, node 1 can reach node 2, and node 2 can reach node 3. The black boxes show when a unit has failed to receive a valid response before timing out. The blue boxes show when a unit is attempting a communication it believes has no relays. The orange boxes show when a unit is attempting a communication it believes has 1 relay. The purple boxes show when a unit is attempting a communication it believes has 2 relays. The green box indicates search completion.

The outcomes of the recursive completion of this logic will result in either a valid path to the destination being discovered or losing track of the destination unit. Should a unit be lost, the network will reform itself by repeating the logic until the unit is rediscovered. The only scenario in which a functioning unit can be irrecoverably lost is one in which no other unit can see it. Once the unit is visible again, a reformat will rediscover the lost unit. Figure 10 shows a timeline of searching for unit 3 in the case when no connections are possible other than B to 1, 1 to 2, and 2 to 3.

This logic will take considerable time to make discoveries, and once lost, it will need to both check each and if the connection times out, see if a relayed connection could be established and continue to search from the relay’s perspective waiting longer for not only the connection to be searched for, but be relayed back. Making the search even more thorough, this will attempt to continue relaying until and repeating searches until all options have been exhausted. In case a system lag causes a failed communication, each step in this process is repeated once before moving to the next step. More efficient algorithms could make searching faster, but this algorithm will leave no connection left unchecked, including all relay connections.

A timeout time of 150 bits with no response was chosen to allow for a theoretical 4-node-long connection, the time it takes to travel to the destination and back to the source. Each failed connection adds 150 bits (at 10 kHz, that makes 15 ms) to the connection time. Adding that each will be tried twice, then relayed and tried twice, this algorithm, in combination with this system, can expect results in the range of seconds. An intelligent search can eliminate the majority of this time; however, this will provide good results to compare against each other and to test the differences when obstructed or forced to change.

## 4. Results and Evaluations

### 4.1. Experiment Setup

To test the effectiveness of this network to maintain connectivity in a swarm configuration, an experiment was devised in which 4 units were placed in waterproof crude submersibles. These submersibles were fitted in such a way that the LED would reflect in as wide an angle as possible to allow for a message to be received as openly as possible. Due to the possibility of receiving its own signal, the device will ignore any signals with its own ID as the sender (as would be the case while a relayed message is being sent or while waiting for a confirmation), but otherwise, a unit cannot send and receive a message at the same time.

**Primary Test Setup:** The first test will demonstrate its ability to rapidly adapt to changing configurations. The 4 units will be placed in direct network configurations, consisting of 2 members, 3 members, and then 4 members, to establish a baseline time. The unit at the furthest point in the network will be required to verify 500 **PING**s from the buoy. Once the baseline is established, the units will be placed in a configuration where a preferred 2-member communications is underway. This communication will be manually disrupted by blocking the line of sight, forcing the network to reconfigure into a 3-member configuration. This will be timed to determine if the network can reconfigure in an appropriate timeframe. Another congregation will see a 3-member network have its communication obstructed, such that it must reconfigure to a different 3-member configuration. This should have a similar time to an ordinary 3-member configuration.

**Secondary Test Setup:** A second test demonstrating that this network can function underwater in a similar amount of time as in air will be performed. It is already well established that this kind of communication is possible [1,2,3,4,5,6,7], but showing these units’ ability is vital if this type of swarm is to be developed. A unit will be placed inside a housing and will be placed underwater. This unit will be made a part of a 2-member network. This unit will receive exactly 100 **PING** message from the buoy. The buoy will track how many of these managers are properly returned by the unit. This test will be performed 10 times, both in air and water. The comparison of the packet failure rate will provide insight into the differences in performance in different media. The performance in each medium should be similar.

In every test, the buoy will be communicating via a Wi-Fi connection to a lab computer to report back the results of the tests. This is the only unit that is permitted to communicate via any means other than blue light. It will initiate each test by messaging the furthest and/or intended unit **PING** until it receives 500 **Affirmative** replies from that unit in the case of the primary test. If it does not receive the **Affirmative** message from the desired unit before trimming out, or it does not believe it can see the unit, it will recursively execute the logic seen in Figure 9 with any unit it can see until it has established a connection. In the case of the secondary test, exactly 100 **PING** message will be sent and only the **Affirmative** response that should accompany every **PING** will be counted as a successful message. The node-to-node distances were not specifically measured, but were made short in order to minimize both the likelihood of an unintended path being discovered and the impact of multi-path reflections. To further ensure that multi-path reflections were a negligible factor, during all testing physical barriers were installed around the units to prevent any unintended (or not currently being tested for) paths from being discovered both as a network or as individual beams of light. The only point at which any of these physical barriers were adjusted was during the reconfiguration tests, where the relevant barriers were quickly adjusted to allow the network to discover the new intended path and prevent the old path from working.

These tests will allow conclusions to be drawn about how this network will perform in a general arbitrary configuration. The 4 total nodes at play will allow the network to demonstrate its ability to effectively communicate in cases where multiple paths are viable and adapt without user input in cases where preferred communication is interrupted, while allowing the sender and receiver nodes to remain in the network. Functionality demonstrated here is expandable to any number of nodes, but can not be proven with any fewer than 4 nodes. With 4 nodes, all failure modes of an adaptive relay network with a fixed sender and receiver may be experienced. With 2 possible connection types and 2 possible failed connection types, these modes are successful connection, successful relay, missed connection, and missed relay. Including more nodes will increase the complexity of the recursion, increase the time to communicate even further, and multiply the number of possible configurations, but will not introduce any new failure mode that can not be modeled by 4 nodes. By using the last successful node as the sender, and the intended next node as the destination, no new states may be observed. Depending on the number of available nodes, the missed relay state may be repeated multiple times, but each is effectively an identical state. Though this condensing of previous successful and future indeterminate nodes, it can be determined that it takes a minimum of 4 nodes to experience all possible states. It can then be safely inferred that any state observed in the network can be observed in larger networks, but larger networks can’t display an error that can’t be observed here.

Though the anticipated data rate of this system is on par with current solutions, systems that employ better electronics will be able to take full advantage of the higher data rate of optics. All this system needs to demonstrate to prove feasibility is overcoming changing line of sight limitations without a large loss of relative data rate.

### 4.2. Test Data

Each test is denoted by its configuration or medium. A configuration **B-1** is interpreted as a network configuration consisting of unit **B**, the buoy, and unit **1**. An arrow **->** indicates that a change of configuration has been performed. A configuration **B-1 -> B-2** is interpreted as a network configuration consisting of unit **B**, the buoy, and unit **1** that changed to a network consisting of unit **B**, the buoy, and unit **2**.

#### 4.2.1. B-1 Configuration

**Figure 11:**
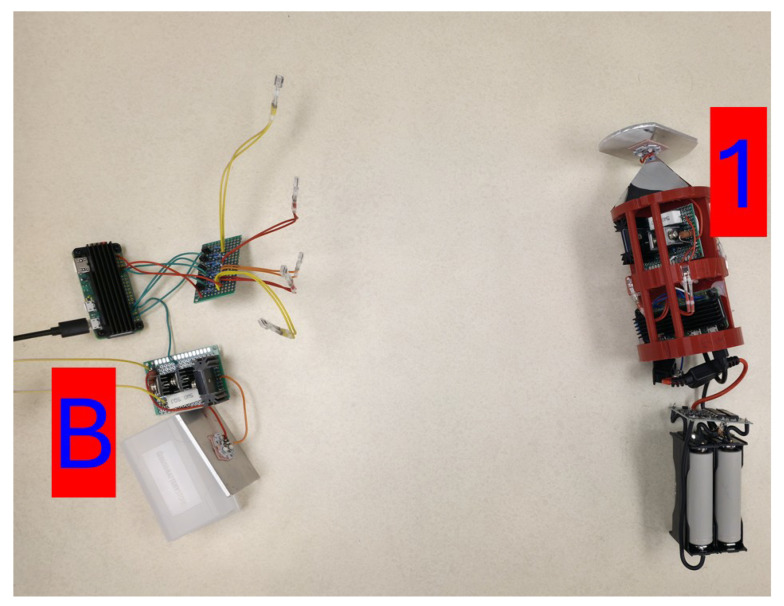
The ‘B-1’ network configuration.

Figure 11 displays a photograph of the Buoy and Unit 1 as they performed 5 trials consisting of 500 **PING** requests. The data recoded by these trials are shown in Table 2.

#### 4.2.2. B-1-2 Configuration

**Figure 12:**
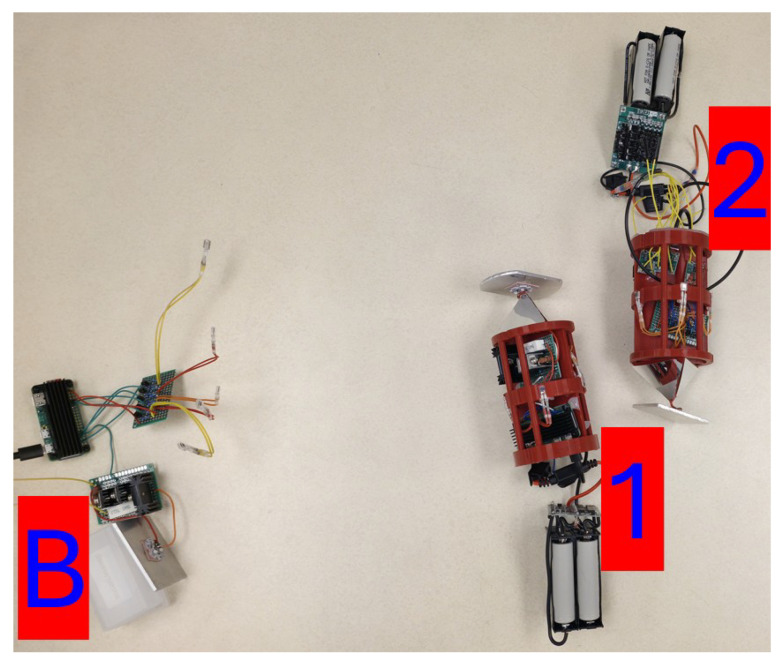
The ‘B-1-2’ network configuration.

Figure 12 displays a photograph of the Buoy, Unit 1, and Unit 2 as they performed 5 trials consisting of 500 **PING** requests. The data recoded by these trials are shown in Table 2.

#### 4.2.3. B-1-2-3 Configuration

**Figure 13:**
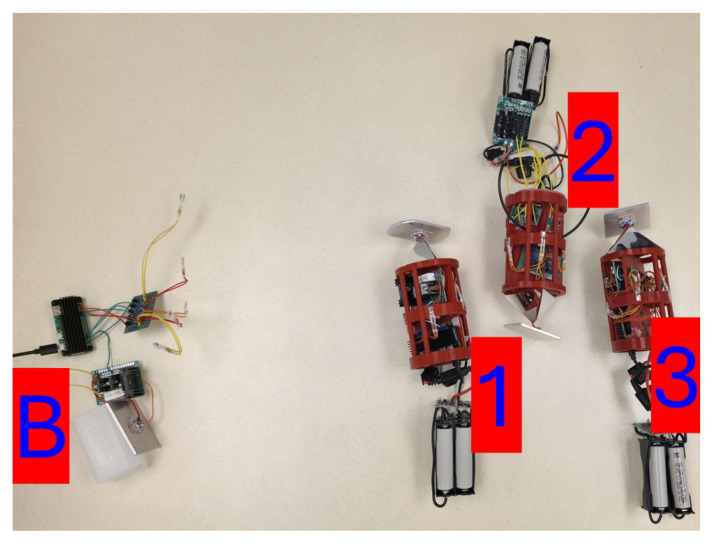
The ‘B-1-2-3’ network configuration.

Figure 13 displays a photograph of the Buoy, Unit 1, Unit 2, and Unit 3 as they performed 5 trials consisting of 500 **PING** requests. The data recoded by these trials are shown in Table 2.

#### 4.2.4. B-1-> B-2-1 Configuration

**Figure 14:**
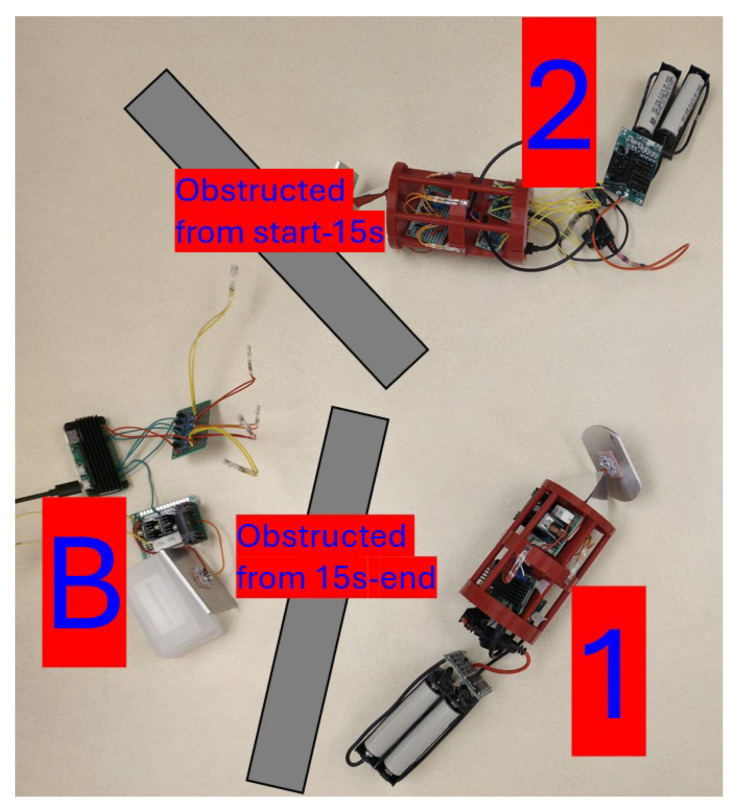
The ‘B-1 -> B-2-1’ network configuration.

Figure 14 displays a photograph of the Buoy, Unit 1, and Unit 2 as they performed 5 trials consisting of 500 **PING** requests. When the recording of these trials started, the network was in a B-1 configuration. The path from the Buoy to Unit 2 was visually obstructed by a piece of non-transparent wood. After 15 s, the path from the Buoy to Unit 1 was obstructed, and the path from the Buoy to Unit 2 was opened. The data recoded by these trials are shown in Table 2.

#### 4.2.5. B-3-2-> B-1-2 Configuration

**Figure 15:**
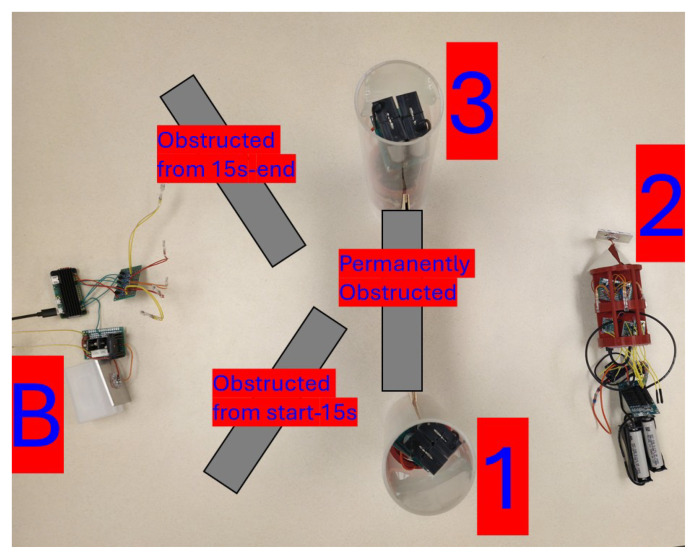
The ‘B-3-2 -> B-1-2’ network configuration.

Figure 15 displays a photograph of the Buoy, Unit 1, Unit 2, and Unit 3 as they performed 5 trials consisting of 500 **PING** requests. When the recording of these trials started, the network was in a B-3-2 configuration. The path from the Buoy to Unit 1 was visually obstructed by a piece of non-transparent wood. After 15 s, the path from the Buoy to Unit 3 was obstructed, and the path from the Buoy to Unit 1 was opened. The data recoded by these trials are shown in Table 2.

#### 4.2.6. Underwater

**Figure 16:**
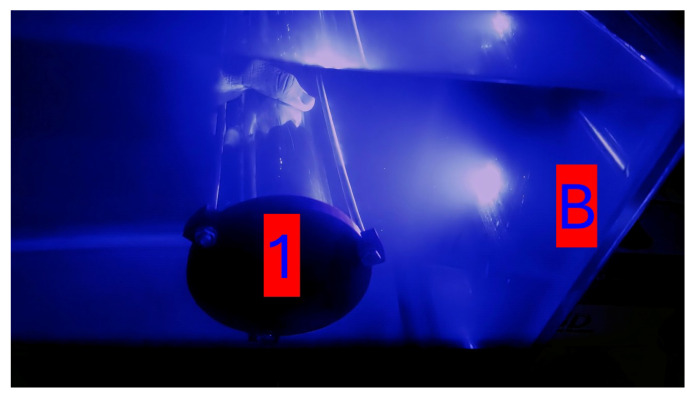
A unit placed underwater.

Figure 16 displays a photograph of the Buoy and Unit 1 as they performed 10 trials consisting of 100 **PING** requests. This trial is an identical configuration to the trial that was performed on the B-1 configurations above water, other than the fact that this test was performed with Unit 1 placed inside the fully assembled case seen in Figure 4 and performed in water. This test was repeated outside of the water to record the in-air success rate data. The data recoded by these trials are shown in Table 3.

### 4.3. Simulation

The primary tests require a baseline to be compared to. Ideally, this system will behave as a generic adaptive relay network with approximately equivalent latency between nodes. To establish this baseline and find the trend that this system is expected to meet, a simulation was run in Java. This simulation followed the same set of guidelines as the real test. In this simulation, each configuration was tested for 500 simulated **PING** requests. As the purpose of this simulation is to provide a baseline, each **PING** was allocated a random variation of up to 50% longer than 20ms. If this **PING** fell within 10% of the maximum variation, this **PING** would be considered a Time-Out and would start over while still counting its original time.

**Figure 17:**
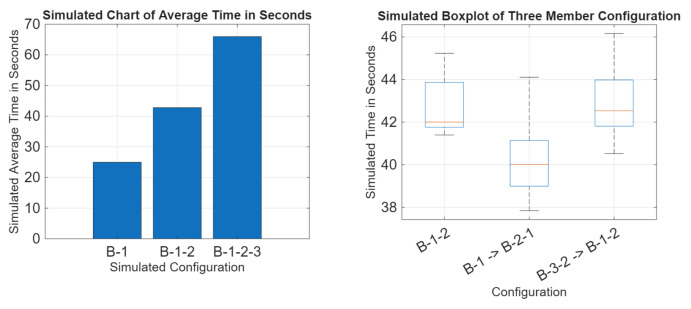
Simulated results data.

Where this model differs in testing methodology is that for the tests that swap configurations after a given time period, this simulation swaps after 100 completions and swaps immediately to simulate a successful network adaptation. The real systems would have to discover the new configuration on their own. While this simulation is not an accurate model of the real system, it does establish a baseline trend for adaptive relay networks of this type. Due to this simulation not being an accurate representation of the real system, the values of the results can not be interpreted as expected values, but how those simulated results compare to each other is the mark that the real system should match. The real data should take a form similar to that of the simulated data if it is acting as an adaptive relay network. Figure 17 displays a comparison of the simulated results.

#### Simulation Analysis

Analyzing Figure 17, the behavior of an adaptive relay network can be established. It can be seen that an increase in the number of nodes leads to an increase in the expected time to communicate, communication times among similarly long network configurations are similar regardless of adaptation, and configurations that change node length see an extension in time towards the longer configuration. If the real data matches this trend, it is established that it is behaving like an adaptive relay network. While this simulation changes configuration at an arbitrary point, if the real network matches this trend, it proves that it can overcome line-of-sight obstructions despite being a free-space optical system.

### 4.4. Collected Real Data

Table 2 shows the complete runtime and average runtime of each configuration for five runs. It summarizes the recorded timing results across multiple network configurations and experimental runs. Each configuration represents a distinct communication/networking path between B and subsequent units, with the average time computed over five independent trials. Table 4 shows a network map to clarify the used communication paths used in each configuration. The results indicate a clear increase in total discovery or cooperative communication time as the network depth increases.

**Figure 18:**
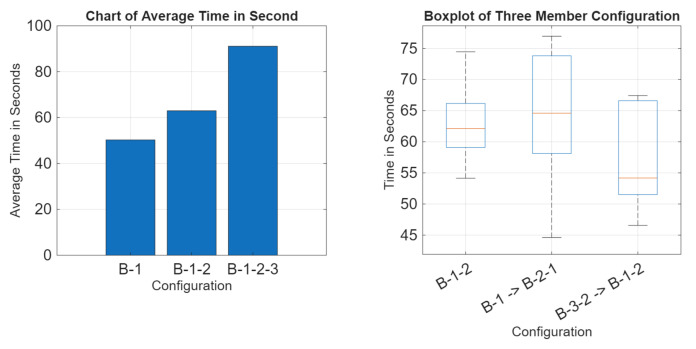
Real results data.

**Figure 19:**
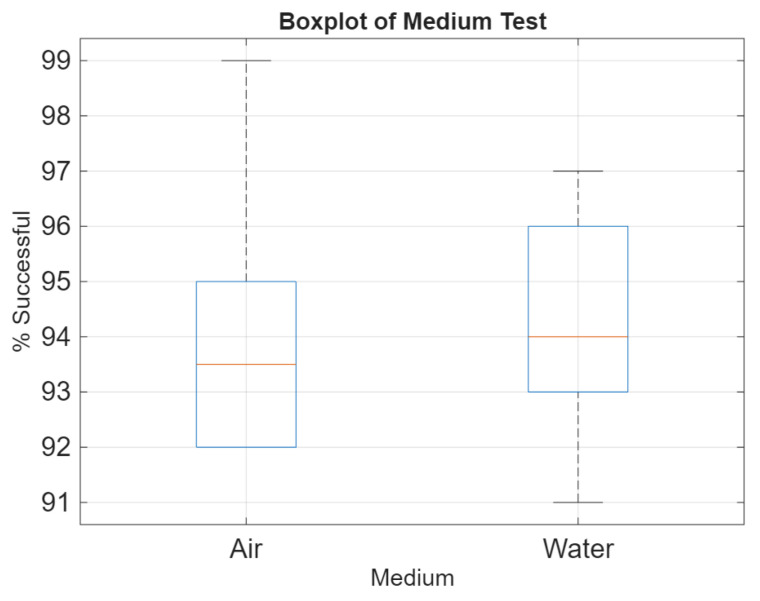
Success rate results.

The results of the real data have been applied to the same graphic as the simulation. The graphic is seen in Figure 18. The success rate data can be seen in Figure 19.

### 4.5. Discussion and Analysis

As seen in Figure 18, the communication times between networks of the same total number of nodes are not greatly affected by reconfiguration. Networks that change the total number of nodes during reconfiguration see an extension in time to communicate towards the longer configuration. Figure 19 confirms that this system performs similarly underwater as in air.

This result is consistent with our simulation. Going from a B-1 to a B-1-2 configuration sees an average of a 25.24% increase in time to communicate, and going from B-1-2 to a B-1-2-3 configuration sees an average of a 44.66% increase in time to communicate. When comparing the data from the B-1 and B-1 underwater configuration, while the water results display a higher average, data overlap is still present. Comparing the B-1-2, B-1 -> B-2-1, and B-3-2, B-1-2 configurations, all tested 3-member networks contain data overlapping. Going from a 2-member network to a 3-member network, as seen in the B-1 -> B-2-1 data, shows that times begin to resemble the 3-member network as opposed to the 2-member times seen in B-1. The more members within an attempt to communicate, the longer it takes to communicate. B-3-2 -> B-1-2 displays a similar time with great data overlap compared with the B-1-2 data. Reconfiguring does not significantly increase the time to communicate.

## 5. Conclusions and Future Work

Free-space optical communications, when paired with an adaptive relay network, have shown an ability to enable autonomous underwater drone swarms by overcoming/compensating for the known system limitations of optical systems, including line-of-sight dependent communication, comparably limited range, obstruction-vulnerable communication, and light interference-vulnerable communication. While the system produced by this study was unable to reach the known data rate capacity of current air-based free-space optical communication systems, a system that is able to reach this speed can plausibly outperform existing wireless systems in terms of data extraction, operation in tight spaces, and operation near propellers, thanks to the lack of a tether. The known theoretical benefits of free-space optical communications systems over alternative systems now appear to be practically attainable in real systems that implement adaptive relay networking. Future underwater drone systems can reasonably consider free-space optical communication as an alternative to acoustic or tether-based communication.

While the use of a microcomputer simplified system development, it is recommended to replace it with a microcontroller or an FPGA board capable of generating arbitrary waveforms at 25 MHz to achieve better real-time performance and signal fidelity. In addition, the units experienced substantially higher internal temperatures than anticipated when enclosed in their sealed, waterproof housings; therefore, enhanced thermal management and improved cooling mechanisms should be considered in future designs. The limitations imposed by the Raspberry Pi are more difficult to overcome due to the nature of having an operating system installed on each unit. Due to all attempted ’sleep’ or ’wait’ commands tested leading to significantly longer than requested wave periods and the lack of a constant frequency arbitrary waveform generator, timing individual signal changes was achieved using a method typically called ’busy waiting’. This method is notoriously inefficient, and our future work will target methods that could avoid its use. Additionally, the units experienced unexpectedly great heating when enclosed, and cooling will be prominent in future work. In future work, we will target the maximum achievable throughput using the LED and photodiode pair. Additionally, the adaptive relay network logic will become more intelligent by performing a more efficient search, utilizing positional data from the swarm. To improve the reliability and spectral efficiency of the system, we will propose combined error coding with modulation solutions in our future work. Other future work will investigate energy efficiency and synchronization in multi-hop optical relaying and develop optimized swarm-based routing strategies that maximize lifetime while preserving the robustness and coverage benefits demonstrated in this study.

## Figures and Tables

**Table 1:** Prior works in underwater FSO.

Reference	Scenario	Cooperation Scheme	Distinctive Feature
Kaushal et al. [21]	Theoretical	AF	Diverse
Li et al. [22]	Simulation	AF/DF	Hyrbid AF/DF
Sharma et al. [23]	Simulation	Best-Relay Selection	Orientation aware
Islam et al. [24]	Simulation	HAFSO	Power savings
Jamali et al. [25]	Simulation	Multi-Hop	Laser based
Theocharidis et al. [26]	Theoretical	AI-Assisted	Optimization focus
Li et al. [27]	Experimental	None	Laser based & real

**Table 2:** Recorded data.

Configuration	Run 1 (s)	Run 2 (s)	Run 3 (s)	Run 4 (s)	Run 5 (s)	Ave. (s)	Stdv. (s)
B-1	48.67	47.76	50.21	53.28	51.49	50.28	2.20
B-1-2	54.15	60.72	62.13	74.45	63.40	62.97	7.34
B-1-2-3	95.23	84.75	86.26	94.74	94.45	91.09	5.13
B-1 -> B-2-1	62.61	44.63	76.96	72.74	64.59	64.31	12.46
B-3-2 -> B-1-2	53.16	54.18	67.42	66.33	46.57	57.53	9.03

**Table 3:** Recorded success data.

Run #	Success	Success
	in Air (%)	in Water (%)
1	95	94
2	92	94
3	93	96
4	93	91
5	94	96
6	92	92
7	97	93
8	92	96
9	95	93
10	99	97
Ave.	94.2	94.2

**Table 4:** Network map. All used valid communication paths are displayed per configuration. Each level can only communicate with the level before and after. Level 1 is always the sender and the highest level is always the destination.

Configuration	Level 1	Level 2	Level 3	Level 4	Changes
B-1	B	1			No
B-1-2	B	1	2		No
B-1-2-3	B	1	2	3	No
B-1 -> B-2-1	B	1			At 15 s
	B	2	1		
B-3-2 -> B-1-2	B	3	2		At 15 s
	B	1	2		

## Data Availability

The original contributions presented in this study are included in the article. Further inquiries can be directed to the corresponding author(s).

## Abbreviations

The following abbreviations are used in this manuscript:

3D	3-Dimensional
AF	Amplify-and-Forward
AI	Artificial-Intelligence
Ave.	Average
BMS	Battery Management System
DF	Decode-and-Forward
FSO	Free-Space-Optics
FPGA	Field-Programmable Gate Array
GPIO	General Purpose Input Output
HAFSO	Hybrid-Acoustic-Free-Space-Optics
ID	Identifier
LED	Light Emitting Diode
Li-ion	Lithium Ion
LoRa	Long-range Radio
LOS	Line of Sight
NRZ	Non-Return-to-Zero
OOK	On Off Keying
PETG+	Polyethylene Terephthalate Glycol (modified)
R-Pi	Raspberry Pi
Stdv.	Standard Deviation
UV	Ultra Violet
Vo	Voltage Output
Wi-Fi	Wireless Fidelity
